# MicroRNA-127 Inhibits the Progression of Melanoma by Downregulating Delta-Like Homologue 1

**DOI:** 10.1155/2020/8523465

**Published:** 2020-01-18

**Authors:** Ping Tian, Ling Tao, Yujun Wang, Xiaobing Han

**Affiliations:** ^1^Xinyang Vocational and Technical College Medical School, Xinyang 464000, China; ^2^Department of Gastroenterology, Xinyang Central Hospital, Xinyang 464000, China; ^3^Department of Oncology, Xinyang Central Hospital, Xinyang 464000, China

## Abstract

**Objective:**

Melanoma is the most common form of skin cancer with low survival rate and poor prognosis. MicroRNAs (miRNAs) have been reported to play essential roles in progression of melanoma. However, the role and mechanism of miR-127 in the process of melanoma remain poorly understood.

**Methods:**

The expressions of miR-127 and delta-like homologue 1 (DLK1) were measured in melanoma tissues and cells by quantitative real-time polymerase chain reaction (qRT-PCR) and Western blot. Cell proliferation and apoptosis were measured by MTT assay, flow cytometry, and Western blot. The interaction between miR-127 and DLK1 was investigated by bioinformatics analysis, luciferase activity assay, and RNA immunoprecipitation (RIP). Murine xenograft model was conducted to investigate the effect of miR-127 on tumor growth in vivo.

**Results:**

miR-127 was inhibited and DLK1 mRNA was enhanced in melanoma tissues and cells. Low abundance of miR-127 in melanoma tissues predicted a poor prognosis and was associated with the malignant clinicopathological features. Overexpression of miR-127 inhibited cell proliferation and induced apoptosis in melanoma cells. Moreover, DLK1 was targeted by miR-127 and its restoration reversed the regulatory effect of miR-127 on the process of melanoma. Besides, the addition of miR-127 suppressed xenograft tumor growth via suppressing DLK1 protein level in nude mice.

**Conclusion:**

miR-127 blocked the development of melanoma by targeting DLK1, providing a novel biomarker for the treatment of melanoma.

## 1. Introduction

Melanoma is one of the most common malignant skin tumors with high incidence and mortality worldwide [1]. Despite the great advance in the treatment of melanoma, including surgery, radiotherapy, chemotherapy, and immunotherapy, the 5-year survival and prognosis remain poor [2]. In recent years, there is a rapid development of targeted drugs and therapeutics for the treatment of melanoma, while effective strategies are limited. Hence, novel biomarkers for prognosis and therapeutics of melanoma are demanded.

MicroRNAs (miRNAs) are a class of small noncoding RNAs, which have essential roles in the progression of many cancers by regulating proliferation, apoptosis, migration, and invasion [3]. Moreover, miRNAs have been reported to have an important impact on the process of melanoma [4]. For example, miR-590-5p suppressed cell proliferation and tumor growth in melanoma by regulating Yes-associated protein 1 (YAP1) [5]. Moreover, miR-143-3p inhibited growth, migration, invasion, and induced apoptosis by targeting cyclooxygenase-2 (COX-2) in melanoma cells [6]. As for miR-127, it has been reported to regulate cell proliferation, migration, invasion, and prognosis of patients by mediating replication initiator 1 (REPIN1) in glioma [7]. Furthermore, miR-127 has been suggested as a tumor suppressor to mediate cell proliferation and senescence by regulating B-cell lymphoma 6 (BCL6) in breast cancer cells [8]. In addition, miR-127 suppresses cell viability, migration, and invasion and contributes to apoptosis in osteosarcoma cells [9]. Besides, miR-127 has been indicated to be ectopic in melanoma patients [10]. However, the roles of miR-127 in melanoma progression and its mechanism remain poorly understood.

Delta-like homologue 1 (DLK1) is one of the transmembrane and secreted proteins in the epidermal growth factor-like homeotic family, which is associated with the oncogenic activity of glioma [11]. Moreover, DLK1 has been reported to play essential roles in the development of atherosclerosis by regulating endothelial proliferation [12]. Besides, it has been indicated that DLK1 facilitates cell proliferation and oncogenic potential of melanoma cells [13]. Intriguingly, bioinformatics analysis provides the putative binding sites of miR-127 and DLK1. Hence, we speculate that DLK1 may be required for miR-127-mediated effect on the progression of melanoma. In the present study, we measured the expressions of miR-127 and DLK1 in melanoma tissues and cells and explored the potential mechanism that underlies miR-127 regulating progression of melanoma in vitro and in vivo.

## 2. Materials and Methods

### 2.1. Patients and Tissue Samples

The melanoma specimens and adjacent normal samples were obtained from 40 patients without the history of radiotherapy, chemotherapy, or related therapy before surgery in Xinyang Central Hospital. All samples were immediately frozen in liquid nitrogen and then stored at −80°C until required. Informed consent was obtained from all participants and the work was conducted under the instructions accepted by the Research Ethics Committee of Xinyang Central Hospital. The patients with an abundance of miR-127 higher than mean value were attributed into the high miR-127 group (*n* = 17), whereas those with miR-127 expression less than mean value were defined as the low miR-127 group (*n* = 23). The clinicopathological characteristics of the patients were shown in Table 1. A follow-up was performed for analysis of a 5-year overall survival rate.

### 2.2. Cell Culture and Transfection

Melanoma cell lines (WM35, SK-MEL-5, SK-MEL-2, A375) and human epidermal melanocytes (HeMa-Lp) were obtained from American Tissue Culture Collection (ATCC, Manassas, VA, USA). All cells were cultured in RPMI-1640 (Gibco, Carlsbad, CA, USA) with 10% fetal bovine serum (FBS, Gibco) and 1% penicillin and streptomycin (Invitrogen, Carlsbad, CA, USA) at 37°C in an incubator with 5% CO_2_ during the study.

The DLK1 overexpression vectors (DLK1), empty vector pcDNA, miR-127 mimic (miR-127), miR-127 inhibitor (anti-miR-127), and negative control (miR-NC or anti-miR-NC) were obtained from GenePharma (Shanghai, China). Transfection was conducted in SK-MEL-2 and WM35 cells using Lipofectamine 3000 (Invitrogen) according to the manufacturer's protocol.

### 2.3. Quantitative Real-Time Polymerase Chain Reaction (qRT-PCR)

Total RNA was isolated from tissues and cells using Trizol reagent (Invitrogen) according to the manufacturer's instructions. The complementary DNA (cDNA) was synthesized by using TaqMan miRNA reverse transcription kit (Applied Biosystems, Foster City, CA, USA) or M-MLV reverse transcriptase (Invitrogen), followed by qRT-PCR with SYBR Green Master Mix (Applied Biosystems). The relative expression of miR-127 or DLK1 was analyzed using 2^−ΔΔCt^ method and normalized to U6 or *β*-actin, respectively. All primers were listed as follows: miR-127 (forward, 5′-AGTCACTGTTAACCAGCCTGCT-3′; reverse, 5′-TTAACTTACGAGGGAGGCAGATGA-3′), U6 (forward, 5′-CTCGCTTCGGCAGCACA-3′; reverse, 5′-AAC GCTTCACGAATTTGCGT-3′), DLK1 (forward, 5′-GCGAGGATGACAATGTTTGCA-3′; reverse, 5′-GGTTCTCCACAGAGTCCGTGAA-3′), *β*-actin (forward, 5′-AGGCACCAGGGCGTGAT-3′; reverse, 5′-GCCCACATAGGAATCCTTCTGAC-3′).

### 2.4. Cell Proliferation

Cell proliferation was analyzed by MTT solution (Thermo Fisher, Wilmington, DE, USA). SK-MEL-2 and WM35 cells were seeded into 96-well plates at a density of 1 × 10^4^ cells per well for 24, 48, or 72 h. Each group was prepared in triplicate. At the ending point, cells were incubated with MTT solution for 4 h. Following the removal of the supernatant, 100 *μ*l DMSO (Thermo Fisher) was introduced to dissolve formazan. The absorbance was measured at 570 nm using a microplate reader (Bio-Rad, Hercules, CA, USA).

### 2.5. Cell Apoptosis

Cell apoptosis was investigated via flow cytometry using Annexin V-FITC/propidium iodide (PI) cell apoptosis detection kit (Sigma, St. Louis, MO, USA). Treated SK-MEL-2 and WM35 cells were collected and resuspended in the binding buffer after being washed with PBS. Subsequently, cells were incubated with Annexin V-FITC and PI for 20 min. The stained cells were detected by using a flow cytometer (BD Biosciences, Franklin Lakes, NJ, USA).

### 2.6. Western Blot

Total protein from cells or tissues was isolated in RIPA buffer containing 1% protease inhibitor (Thermo Fisher) and then quantified using BCA assay kit (Sigma, St. Louis, MO, USA) after the centrifugation. Subsequently, denatured proteins were separated by SDS-PAGE gel electrophoresis and then transferred to polyvinylidene difluoride (PVDF) membranes (Millipore, Billerica, MA, USA). After blocking with 5% nonfat milk for 1 h at room temperature, the membranes were incubated with primary antibodies against B-cell lymphoma-2 (Bcl-2) (ab196495, 1 : 1000 dilution, Abcam, Cambridge, UK), Bcl-2-associated X protein (Bax) (ab53154, 1 : 500 dilution, Abcam), proliferating cell nuclear antigen (PCNA) (ab92552, 1 : 5000 dilution, Abcam), DLK1 (ab210471, 1 : 1000 dilution, Abcam), or *β*-actin (ab8227, 1 : 2000 dilution, Abcam) overnight at 4°C. Following washing with TBST, membranes were hatched with HRP-conjugated secondary antibodies (ab205718, 1 : 10,000 dilution, Abcam) for 2 h at room temperature. The protein signals were captured using films in the dark after incubation with enhanced chemiluminescence (ECL) chromogenic substrate (Thermo Fisher). The relative levels of proteins were investigated by Image Lab software (Bio-Rad).

### 2.7. Luciferase Activity Assay

The putative binding sites of miR-127 and 3′ untranslated regions (3′-UTR) sequences of DLK1 were predicted by TargetScan Release 7.2. Wide-type or mutant luciferase report vectors (DLK1-WT or DLK1-MUT) were generated by using pGL3 vector (Promega, Madison, WI, USA), respectively. Luciferase reporter vectors and miR-127 or miR-NC, along with renilla plasmid were cotransfected in SK-MEL-2 or WM35 cells using Lipofectamine 3000 according to the manufacturer's protocols. After transfection for 48 h, cells were collected and luciferase activity was measured by using a luciferase assay kit (Promega).

### 2.8. RNA Immunoprecipitation (RIP)

RIP assay was conducted by RNA-binding protein immunoprecipitation kit (Millipore) according to the manufacturer's protocols. SK-MEL-2 and WM35 cells transfected with miR-127 or miR-NC were lysed in RIP buffer with anti-Ago2 or IgG bound magnetic beads. After washing with PBS, the beads were incubated with Trizol for qRT-PCR.

### 2.9. Xenograft Model of Melanoma

Four-week-old male BALB/c nude mice were purchased from Vital River Laboratory Animal Technology (Beijing, China). WM35 cells were transfected with lentiviral vectors with miR-127 (LV-miR-127) or miR-NC (LV-miR-NC) constructed by GeneCopoeia (Rockville, MD, USA). Stably transfected WM35 cells (5 × 10^6^ cells) were subcutaneously injected into nude mice (*n* = 7 per group). Tumors volume was examined and calculated with slide calipers by the formula as 0.5 × length × width × height. At 4 weeks after cell implantation, mice were sacrificed and tumor specimens were weighed and collected for qRT-PCR and Western blot. The experiments were approved by the Animal Research Committee of Xinyang Central Hospital.

### 2.10. Statistical Analysis

Data were expressed as the mean ± standard deviation (SD) from three independent experiments. The association between miR-127 and overall survival of patients was investigated by Kaplan–-Meier analysis. Significant difference between groups was analyzed by Student's *t*-test using SPSS 18.0 software (SPSS, Inc., Chicago, IL, USA). *p* values less than 0.05 were regarded as statistically significant.

## 3. Results

### 3.1. miR-127 Was Downregulated in Melanoma Tissues and Cells

**Figure 1:**
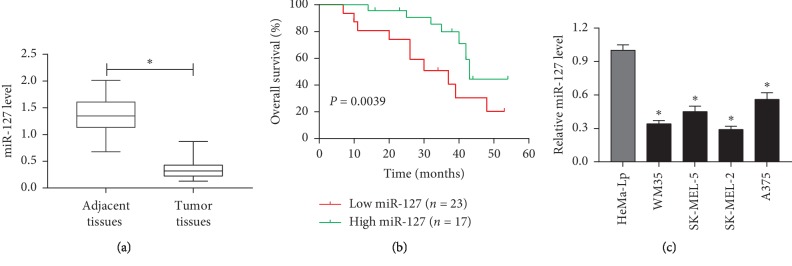
miR-127 was inhibited in melanoma tissues and cells. (a) The expression of miR-127 was measured in tumor and adjacent tissues of melanoma. (b) The overall survival rate was analyzed in low and high miR-127 groups. (c) The abundances of miR-127 were detected in melanoma cells (^*∗*^*p* < 0.05).

To explore the potential role of miR-127 in melanoma, the expression of miR-127 was measured in melanoma tissues and cells. Results showed that miR-127 expression was impaired in melanoma tissues compared with that in adjacent normal samples (Figure 1(a)). Moreover, patients were classified as low and high miR-127 expression groups according to the mean value of miR-127 abundance. The clinicopathological characteristics of the patients were shown in Table 1. The expression of miR-127 was associated with tumor thickness and stage (*p* < 0.05), while there was no association between miR-127 and other characteristics, such as age, gender, and lymph node metastasis (*p* > 0.05). Furthermore, a follow-up was conducted and a lower overall survival rate was shown in the low miR-127 expression group than in the high miR-127 expression group (Figure 1(b)). Besides, the abundance of miR-127 was decreased in melanoma cells, especially in SK-MEL-2 and WM35 cells (Figure 1(c)). Hence, SK-MEL-2 and WM35 cells were used for further experiments.

### 3.2. Overexpression of miR-127 Inhibited Cell Proliferation and Induced Apoptosis in Melanoma Cells

**Figure 2:**
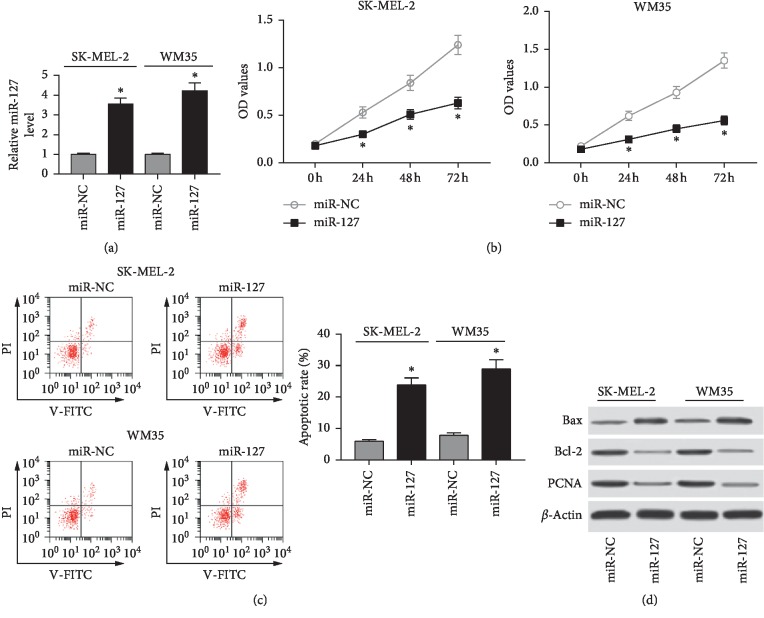
Overexpression of miR-127 suppressed cell proliferation and induced apoptosis in melanoma cells. (a) The transfection efficacy was investigated in SK-MEL-2 and WM35 cells transfected with miR-127 or miR-NC by qRT-PCR. (b) Cell proliferation was measured in SK-MEL-2 and WM35 cells transfected with miR-127 or miR-NC by MTT assay. (c) Cell apoptosis was detected in SK-MEL-2 and WM35 cells transfected with miR-127 or miR-NC by flow cytometry. (d) The abundances of related proteins were examined in SK-MEL-2 and WM35 cells transfected with miR-127 or miR-NC by Western blot (^*∗*^*p* < 0.05).

To probe the role of miR-127 in the progression of melanoma, cell proliferation and apoptosis were investigated in SK-MEL-2 and WM35 cells transfected with miR-127 or miR-NC. As a result, the abundance of miR-127 was effectively elevated in SK-MEL-2 and WM35 cells transfected with miR-127 mimic compared with that in cells treated with miR-NC (Figure 2(a)). MTT assay showed that overexpression of miR-127 significantly limited cell proliferation in SK-MEL-2 and WM35 cells (Figure 2(b)). Moreover, elevated apoptotic rate was displayed in SK-MEL-2 and WM35 cells transfected with miR-127 compared with that in miR-NC group (Figure 2(c)). Besides, the markers expressions of proliferation and apoptosis were measured at the protein level in SK-MEL-2 and WM35 cells. Results showed that the addition of miR-127 induced Bax expression while inhibited Bcl-2 and PCNA protein levels in SK-MEL-2 and WM35 cells (Figure 2(d)).

### 3.3. DLK1 Was a Target of miR-127

**Figure 3:**
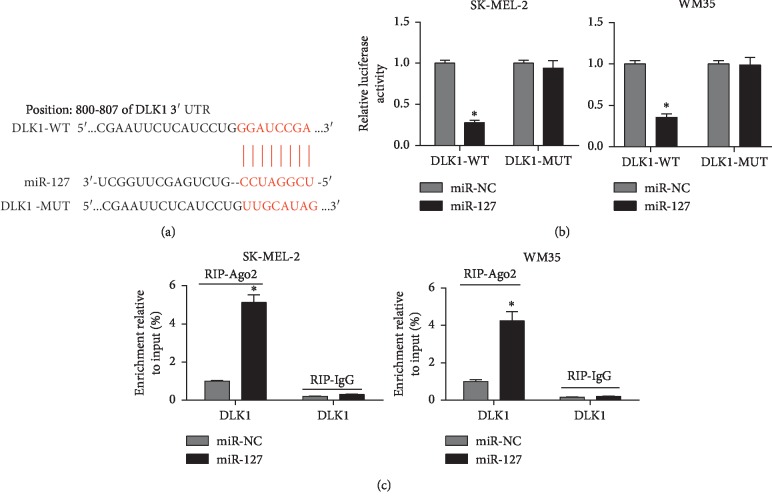
DLK1 was a target of miR-127. (a) The putative binding sites of miR-127 and DLK1 were predicted by TargetScan Release 7.2. (b) Luciferase activity was analyzed in SK-MEL-2 and WM35 cells cotransfected with miR-127 or miR-NC and DLK1-WT or DLK1-MUT. (c) Ago2 RIP assay was conducted in SK-MEL-2 and WM35 cells transfected with miR-127 or miR-NC (^*∗*^*p* < 0.05).

To elucidate the potential mechanism that underlies miR-127-mediated progression of melanoma, one promising target is expected. Bioinformatics analysis provided the putative binding sites of miR-127 and DLK1 by TargetScan Release 7.2 (Figure 3(a)). To identify the prediction, luciferase activity and RIP assay were performed in melanoma cells, respectively. Results showed that overexpression of miR-127 decreased luciferase activity in SK-MEL-2 and WM35 cells transfected with DLK1-WT, whereas the efficacy was lost in response to DLK1-MUT (Figure 3(b)). Moreover, the DLK1 mRNA enriched by Ago2 was obviously enhanced in SK-MEL-2 and WM35 cells transfected with miR-127 compared with that in miR-NC group, while IgG showed little efficacy of enrichment (Figure 3(c)).

### 3.4. DLK1 Was Upregulated in Melanoma Tissues and Cells

**Figure 4:**
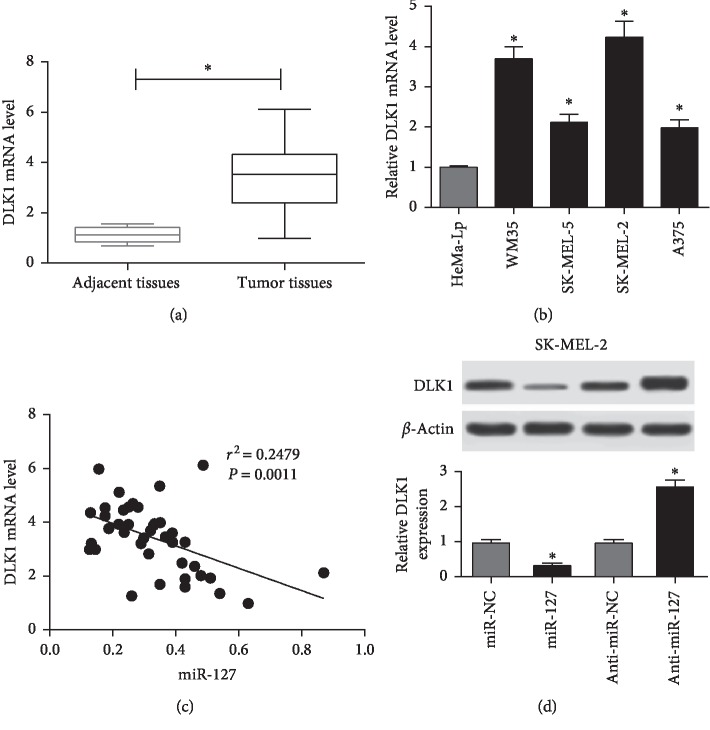
DLK1 was upregulated in melanoma tissues and cells. (a) The mRNA expression of DLK1 was measured in tumor and adjacent tissues of melanoma. (b) The abundances of DLK1 mRNA were detected in melanoma cells. (c) The correlation analysis of miR-127 and DLK1 mRNA was performed in tumor tissues of melanoma patients. (d) The expression of DLK1 protein was measured in SK-MEL-2 cells transfected with miR-NC, miR-127, anti-miR-NC, or anti-miR-127 (^*∗*^*p* < 0.05).

Seeing that DLK1 was targeted by miR-127, the expression of DLK1 was detected in melanoma tissues and cells. Results showed that a great increase of DLK1 mRNA level was indicated in melanoma tissues compared with that in adjacent samples (Figure 4(a)). Similarly, the mRNA expression of DLK1 was also increased in melanoma cells compared with that in HeMa-Lp cells (Figure 4(b)). Moreover, the DLK1 mRNA level was negatively correlated with miR-127 abundance in melanoma patients (*r*^2^ = 0.2479, *p*=0.0011) (Figure 4(c)). Besides, the effect of miR-127 on DLK1 protein expression was investigated in SK-MEL-2 cells. Western blot analysis uncovered overexpression of miR-127 impeded DLK1 protein level and knockdown of miR-127 conferred DLK1 expression (Figure 4(d)).

### 3.5. DLK1 Was Required for miR-127-Mediated Proliferation and Apoptosis in Melanoma Cells

**Figure 5:**
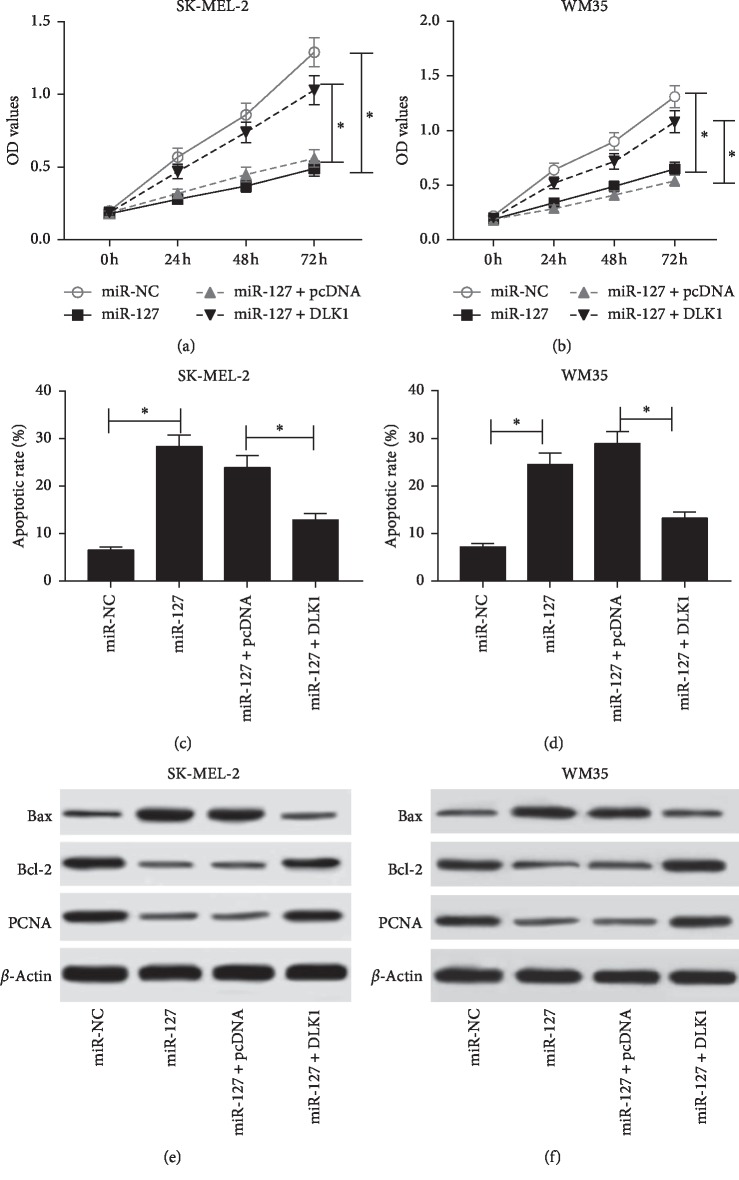
DLK1 was required for miR-127-mediated progression of melanoma. (a and b) Cell proliferation was measured in SK-MEL-2 and WM35 cells cotransfected with miR-127 + pcDNA or miR-127 + DLK1 by MTT assay. (c and d) Cell apoptosis was detected in SK-MEL-2 and WM35 cells cotransfected with miR-127 + pcDNA or miR-127 + DLK1 by flow cytometry. (e and f) The abundances of related proteins were examined in SK-MEL-2 and WM35 cells cotransfected with miR-127 + pcDNA or miR-127 + DLK1 by Western blot (^*∗*^*p* < 0.05).

To validate whether DLK1 was involved in miR-127-mediated progression of melanoma, melanoma cells were transfected with miR-NC, miR-127, miR-127 + pcDNA, or miR-127 + DLK1. Restoration of DLK1 protected cell proliferation from miR-127 in SK-MEL-2 and WM35 cells compared with the treatment of pcDNA (Figures 5(a) and 5(b)). Moreover, overexpression of DLK1 reversed miR-127-induced apoptosis in SK-MEL-2 and WM35 cells (Figures 5(c) and 5(d)). Besides, the upregulation of DLK1 overturned the regulatory effect of miR-127 on the protein abundances of Bax, Bcl-2, and PCNA in SK-MEL-2 as well as WM35 cells (Figures 5(e) and 5(f)).

### 3.6. Addition of miR-127 Suppressed Xenograft Tumor Growth by Regulating DLK1 In Vivo

**Figure 6:**
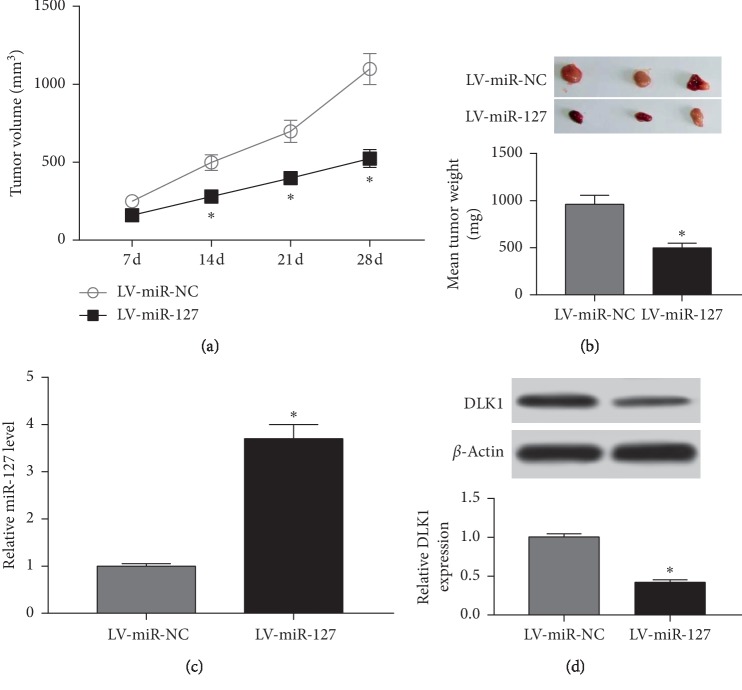
The addition of miR-127 decreased xenograft tumor growth by regulating DLK1 in vivo. (a) Tumor volume was measured every week. (b) Tumor weight was detected at ending point. (c) The expression of miR-127 was examined in tumor tissues. (d) The abundance of DLK1 protein was measured in tumor tissues (^*∗*^*p* < 0.05).

To further investigate the mechanism, a xenograft model was established in vivo. Stably transfected WM35 cells were introduced into nude mice by subcutaneous injection. Results showed that overexpression of miR-127 significantly inhibited tumor volume compared with the treatment of miR-NC (Figure 6(a)). Moreover, tumor weight was evidently decreased in the miR-127 group compared with that in the miR-NC group (Figure 6(b)). Besides, the expressions of miR-127 and DLK1 protein were measured in collected tumor tissues. A strong increase of miR-127 level was indicated in the miR-127 group compared with that in the miR-NC group (Figure 6(c)). However, an obvious inhibition of DLK1 protein abundance was shown in the miR-127 group (Figure 6(d)).

## 4. Discussion

Marked improvements have been suggested to contribute to the prognosis and therapeutics of melanoma patients [14]. However, melanoma remains one of the lethal cancers because of diagnosis at an advanced stage. Displacement of miRNAs presented novel therapeutic targeting opportunities for the treatment of melanoma [15]. In the present study, we investigated the role of miR-127 in the progression of melanoma and provided the first integrative view that miR-127 inhibited cell proliferation and induced apoptosis in melanoma cells by targeting DLK1.

miRNAs have been reported to function as promising biomarkers for diagnosis and prognosis of melanoma [16]. For instance, miR-378 addressed epithelial mesenchymal transition, migration, and invasion by suppressing forkhead box N3 (FOXN3) through regulating Wnt/*β*-catenin pathway [17]. Moreover, miR-331 has been reported to inhibit cell proliferation and invasion by targeting astrocyte-elevated gene-1 (AEG-1) in melanoma cells [18]. Besides, miR-497-5p, miR-195-5p, and miR-455-3p have been regarded as tumor suppressors via blocking human telomerase reverse transcriptase (hTERT) in melanoma [19]. miR-127, as a novel miRNA, has been reported to be involved in the progression of many cancers, such as osteosarcoma, breast cancer, hepatocellular carcinoma, and gastric cancer [9, 20–22]. Moreover, these reports suggested that miR-127 might be regarded as a tumor suppressor in many cancers. Notably, the available evidence has indicated that miR-127 was downregulated in melanoma [10]. Similarly, we also found that miR-127 abundance was impaired in melanoma tissues and cells. Moreover, results showed that low miR-127 expression was associated with tumor thickness, stage as well as low overall survival rate in patients. These data suggested that miR-127 might play an essential role in the progression of melanoma. Cell proliferation and apoptosis have been reported to be key processes of cancer development, characterized by potential targets of proliferation-related protein PCNA, antiapoptotic protein Bcl-2, and proapoptotic protein Bax [23, 24]. However, the exact effect of miR-127 on cell proliferation and apoptosis remains elusive. In this study, we indicated that overexpression of miR-127 inhibited cell proliferation and promoted cell apoptosis. However, a poor understanding of the underlying mechanism allows miR-127 to participate in the progression of melanoma.

Usually, functional miRNAs have been known to regulate the progression of cancer by mediating special targets [25]. Accruing literature has revealed many potential targeted genes of miR-127 in varying cancers. For example, miR-127 affected cell proliferation, migration, invasion, and survival of patients in glioma by targeting REPIN1 [7]. Moreover, miR-127 protected against LPS-induced inflammatory injury via regulating programmed cell death 4 (PDCD4) in PC-12 cells [26]. In addition, miR-127 inhibited tumor growth via targeting cytochrome c oxidase assembly factor 1 homologue (COA1) and protein disulfide isomerase family A, member 6 (PDIA6) in giant cell tumor of bone [27]. Besides, miR-127 suppressed cell proliferation, migration, and tumorigenicity by targeting formin-like 3 (FMNL3) in human esophageal squamous cell carcinoma [28]. Notch pathway has been regarded as antiangiogenesis strategy by reducing tumor growth in melanoma [29]. DLK1 has been indicated as a negative regulator of Notch, which played essential roles in the proliferation and differentiation of ameloblasts [30]. Moreover, DLK1 has been suggested to be positively associated with cell growth in melanoma cells [13]. However, there is no direct evidence in support of the interaction between miR-127 and DLK1. In this study, we first validated the association of miR-127 and DLK1 and indicated that restoration of DLK1 reversed the regulatory effect of miR-127 on cell proliferation and apoptosis in melanoma cells. Besides, the preclinical experiment was responsible for the further understanding of role and mechanism in melanoma [31]. Hence, we established melanoma cells xenograft model in vivo and suggested that miR-127 suppressed tumor growth by regulating DLK1 in mice.

In summary, miR-127 was downregulated and DLK1 was upregulated in melanoma tissues and cells. Moreover, the addition of miR-127 suppressed cell proliferation and promoted cell apoptosis in melanoma. Furthermore, DLK1 was directly targeted by miR-127 and its overexpression abated the effect of miR-127 on the progression of melanoma. Besides, the accumulation of miR-127 decreased tumor growth by suppressing DLK1 in vivo. These findings suggested that miR-127 inhibited melanoma development by targeting DLK1, indicating miR-127 as a promising avenue for the treatment of melanoma.

## Figures and Tables

**Table 1:** Relationship between miR-127 level and clinicopathological characteristics.

Clinicopathological feature	Number	miR-127 level		*p* value
		Low *n* (%)	High *n* (%)	
Age				>0.05
<55 years	24	14 (58.3%)	10 (41.7%)	
≥55 years	16	9 (56.3%)	7 (43.7%)	
Gender				>0.05
Female	22	13 (59.1%)	9 (40.9%)	
Male	18	10 (55.5%)	8 (44.5%)	
Tumor thickness				<0.05
>10 mm	30	21 (70%)	9 (30%)	
≤10 mm	10	2 (20%)	8 (80%)	
Tumor stage				<0.05
I/II	14	6 (42.9%)	8 (57.1%)	
III	26	17 (65.4%)	9 (34.6%)	
Lymph node metastasis				>0.05
Yes	23	13 (56.5%)	10 (43.5%)	
No	17	10 (58.8%)	7 (41.2%)	

## Data Availability

The data used to support the findings of this study are available from the corresponding author upon request.
